# Mortality trends in suicide among pediatric and adolescent patients aged 15–24 years in Mississippi, 2012–2022

**DOI:** 10.1002/pdi3.2511

**Published:** 2024-12-05

**Authors:** Elizabeth Jones

**Affiliations:** ^1^ Department of Epidemiology & Biostatistics Jackson State University Jackson MS USA

**Keywords:** mortality, prevention, suicide

## Abstract

Due to the lack of studies examining suicide trends and its implications on pediatric populations, this study aimed to address the gap in research and to identify the magnitude and the impact of suicide by exploring trends in suicide among Mississippians from 2012 to 2022. The study uses data from the Mississippi Statistically Automated Health Resource System, which is an online database with data collected from vital statistics. Joinpoint regression models were used to calculate annual percentage change (APC) and average annual percentage change (AAPC) as an indicator of trends. The overall age‐adjusted suicide rate increased from 9.4 deaths per 100,000 in 2012 to 10.8 deaths per 100,000 in 2022 for pediatric and adolescent patients aged 15–24 years (14.9% increase). There are upward trends for females (AAPC, 6.33%, 95% CI, −0.82%–16.82%), Blacks (AAPC, 7.72%, 95% CI, 2.19%–16.47%), and other races (AAPC, 7.59%, 95% CI, −0.83%–21.47%). Males had a downward trend from 2015 to 2022 (APC, −1.46%, 95 CI, −14.05%–1.35%). Whites also had a downward trend from 2017 to 2022 (APC, 4.74%, 95% CI, −15.42% to −0.96%). This study identified an overall increase in suicide. However, trends varied by gender, race, and age. Based on the findings, Mississippi needs more initiatives aimed toward equitable prevention of suicide among youth and the implementation of gun control policies. By implementing these measures, Mississippi could tremendously benefit and improve mental health outcomes and reduce suicide within the state.

## Graphical abstract

There are upward trends for females, Blacks, other races. Although males had an upward trend from 2012 to 2015, males had a downward trend from 2015 to 2022. Whites also experienced similar trends. Although Whites had an upward trend from 2012 to 2017, they had a downward trend from 2017 to 2022.

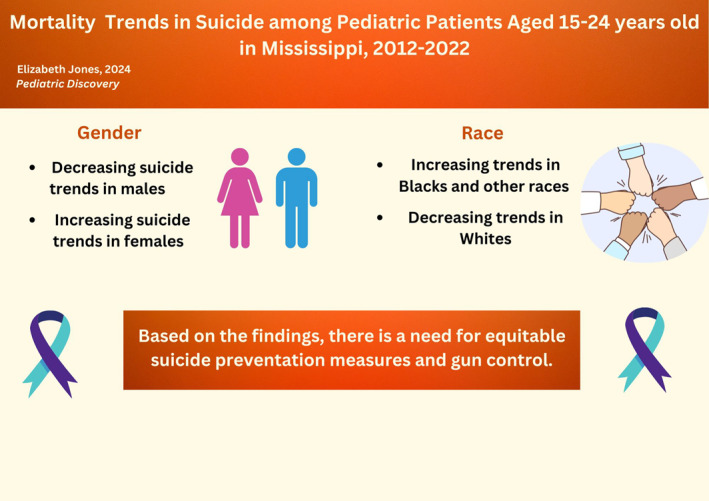

## 1 INTRODUCTION

Suicide is a major public health issue. Globally, more than 700,000 people die by suicide each year.^1^ In 2023, suicide was the fourth leading cause of death worldwide for pediatric and adolescent patients between the ages of 15 and 29 years.^1^ Suicide distributions and patterns highlight major disparities based on sex and age.^2^ Among the 49,449 suicide deaths in the United States in 2022, 79.4% occurred in males, and almost 50% occurred in individuals less than 45 years old.^3^ Youth aged 15–24 years were only included in this study because of the increased rate of suicide occurring within this age range, which signifies a need to identify and address the risk of suicide within this age group.

Various epidemiological studies have shown an increasing trend in suicide in recent years. However, Mississippi's suicide rates are higher than national rates. In 2023, suicide was the third leading cause of death in pediatric and adolescent patients aged 15–24 years.^4^ Examining trends in suicide based on gender, race, and age is important and serves as a tool to bring awareness to the risk of suicide for pediatric and adolescent patients. Despite the importance of the topic, few studies have examined trends in suicide in Mississippi among pediatric and adolescent patients between the ages of 15 and 24 years. To address the gap in research, this study explored the annual percentage change (APC), and average annual percentage change (AAPC) in age‐adjusted suicide rates among Mississippians aged 15–24 years from 2012 to 2022.

## 2 METHODS

### 2.1 Data collection

Data were exported from the Mississippi Statistically Automated Health Resource System (MSTAHRS). The database contains suicide surveillance data from Mississippi. Both the calculation of standard error in SAS Academics Studio and the preparation of MS Excel/text files were used to create mortality models and trends in Joinpoint regression software. Stratification was used to separate the sample into subgroups (gender, race, and age groups).

### 2.2 Statistical analysis

Age‐adjusted rates and frequencies were extracted from MSTAHRS. Standard error was calculated using SAS Studio.^5^ The APC and AAPC of mortality rates in various subgroups were calculated using the US Surveillance, Epidemiology, and End Results (SEER) Joinpoint regression program version 5.0.^6^ Joinpoint regression describes trends and significant changes in trends.

Based on the Bayesian information criterion, the empirical quantile method was used to identify the significant best fit line for trend 1 and trend 2.^7^ *P*‐value was not calculated based on the method. However, each model tested for significance and listed the results for significance. Confidence intervals were calculated for APC and AAPC.

## 3 RESULTS

### 3.1 Understanding age adjustment, APC and AAPC

Age‐adjusted mortality is the rate of deaths in a population regardless of age. Increases and decreases in age‐adjusted mortality signify that deaths in a population have increased or decreased regardless of age. Age‐adjusted mortality rates were used in this study because using age‐adjusted rates reduce the risk of bias.

APC and AAPC are statistical tools used in joinpoint regression, which is used for assessing statistical trends. APC is used to depict the change within a segment of the years of the study. The AAPC is used to depict the change within the total years of the study. A significant APC and/or AAPC means that there is a valid change in the trend that is being evaluated. APC and AAPC were selected as a statistical approach for this study because joinpoint regression is a more thorough means of identifying trends using reliable models.

### 3.2 Calculating APC and AAPC

#### 3.2.1 APC

APC assumes the change at a constant percentage of the rate of the previous year to predict outcomes. Therefore, the following regression model is used to estimate the APC for a series of data:

$\log\left(R_{y}\right)=b_{0}\left(slope\right)+b_{1}\left(y-intercept\right)$, where $\log\left(R_{y}\right)$ is the natural log of the rate in year “*y*”.

The APC from year “*y*” to year “*y* + 1”.

 = $\left[\frac{R_{y+1}-R_{y}}{R_{y}}\right]*100$

 = $\frac{\left\{e^{b_{0}+b_{1}\left(y+1\right)}-e^{b_{0}+b_{1}\left(y\right)}\right\}}{e^{b_{0}+b_{1}\left(y\right)}}*100$

 = $\left(e^{b_{1}}-1\right)*100$

#### 3.2.2 AAPC

The AAPC is a weighted average of the slope coefficients of the underlying Joinpoint regression model with the weights equal to the length of each segment over the interval. If we denote *b*_*i*_ as the slope coefficient for the *i*th segment with *i* indexing the segments in the desired range of years, and *w*_*i*_ as the length of each segment in the range of years, then:

$$AAPC=\left\{\exp\left(\frac{\sum w_{1}b_{1}}{\sum w_{1}}\right)-1\right\}*100$$

Overall Suicide Findings for Pediatric and Adolescent Patients Aged 15–24 years.

Between 2012 and 2022, Mississippi had a total of 577 deaths from suicide. The source population for suicide rates were approximated from 2012 to 2022 and was obtained from Mississippi vital statistics. Majority of the cases occurred in males (87.2%), Whites (71.9%), and age group 5–44 years (48.8%) (Table 1). The overall age‐adjusted suicide rate increased from 9.4 deaths per 100,000 in 2012 to 10.8 deaths per 100,000 in 2022 for pediatric and adolescent patients aged 15–24 years (14.9% increase) (Table 1).

**TABLE 1:** Number of suicides, 2012–2022.

Variables	(*N* = 577)^a^	%
Gender		
Male	503	87.2
Female	74	12.8
Race		
Black	143	24.8
White	415	71.9
Other	19	3.3
Age group		
5–14 years	56	1.2
15–24 years	577	12.5
25–34 years	811	17.6
35–44 years	800	17.4
45–54 years	789	17.2
55–64 years	690	15.0
65–74 years	477	10.4
75–84 years	286	6.2
85+ years	112	2.4

*Note:* Table 1 shows the number and percentage of suicide by gender, race, and age.

^**a**^Total number of cases reported among 15–24 years.

### 3.3 Suicide by gender in 15–24‐year‐olds

From 2012 to 2022, the age‐adjusted suicide rate among males increased by 13% (2.3 deaths per 100,000 to 2.6 deaths per 100,000), with an average annual increase of 3.29% (AAPC, 3.29%, 95% CI, −0.74%–7.86%). The age‐adjusted suicide rate among females increased by 33.3% (0.3 deaths per 100,000 to 0.4 deaths per 100,000), with an average annual increase of 6.33% (AAPC, 6.33%, 95% CI, −0.82%–16.82%) (Table 2).

**TABLE 2:** Trends in suicide, 2012–2022.

Characteristic	No. of cases (age‐adjusted rates)		AAPC (95% CI)	Trend segment 1 (95% CI)		Trend segment 2 (95% CI)	
	**2012**	**2022**	2012–2022	Years	APC	**Years**	**APC**
Gender							
Male	36 (2.3)	39 (2.6)	3.29 (−0.74 to 7.86)	2012–2015	15.28 (3.78 to 51.85)	2015–2022	−1.46 (−14.05 to 1.35)
Female	5 (0.3)	6 (0.4)	6.33 (−0.82 to 16.82)	2012–2022	6.33 (−0.82 to 16.82)		
Race							
Black	7 (0.5)	8 (0.6)	7.72 (2.19 to 16.47)	2012–2022	7.72 (2.19 to 16.47)		
White	33 (2.0)	33 (2.0)	0.79 (−1.67 to 3.49)	2012–2017	6.6 (2.66 to 20.76)	2017–2022	−4.74 (−15.42 to −0.10)
Other	1 (1.1)	4 (3.1)	7.59 (−0.83 to 21.47)	2012–2022	7.59 (−0.83 to 21.47)		
Age group (yrs)							
5–14	2 (0.5)	8 (2.1)	5.47 (−0.23 to 14.33)	2012–2022	5.47 (−0.23 to 14.33)		
15–24	41 (9.4)	45 (10.8)	2.52 (−0.10 to 6.64)	2012–2022	2.52 (−0.10 to 6.64)		
25–34	64 (16.5)	84 (22.3)	5.09 (2.40 to 8.65)	2012–2022	5.09 (2.40 to 8.65)		
35–44	83 (22.4)	65 (17.9)	−0.05 (−3.57 to 3.98)	2012–2022	−0.05 (−3.57 to 3.98)		
45–54	90 (22.4)	66 (18.8)	−1.03 −(−4.81 to 2.67)	2012–2022	−1.03 −(−4.81 to 2.67)		
55–64	57 (15.7)	55 (14.8)	−1.72 (−7.09 to 3.38)	2012–2022	−1.72 (−7.09 to 3.38)		
65–74	27 (11.6)	49 (16.2)	3.13 (−2.33 to 9.88)	2012–2022	3.13 (−2.33 to 9.88)		
75–84	27 (21.7)	30 (19.6)	2.74 (−1.02 to 7.36)	2012–2022	2.74 (−1.02 to 7.36)		
85+	11 (23.3)	17 (32.8)	2.16 (−7.41 to 12.65)	2012–2022	2.16 (−7.41 to 12.65)		

*Note:* Table 2 shows age‐adjusted rates, AAPC, and APC by gender, race, and age.

Abbreviations: AAPC, Average Annual Percentage Change; APC, Annual Percentage Change.

The trends in males consisted of 2 segments: a significant APC of 15.28% (95% CI, 3.78%–51.85%) in the first segment (2012–2015), and a significant APC of −1.46% (95 CI, −14.05%–1.35%) in the second segment (2015–2022). The trends in females consisted of 1 segment: a significant APC of 6.33% (95% CI, −0.82%–16.82%) in the segment (2012–2022). (Table 2).

### 3.4 Suicide by race in 15–24‐year‐olds

From 2012 to 2022, the age‐adjusted suicide rate among Blacks increased by 20% (0.5 deaths per 100,000 to 0.6 deaths per 100,000), with an average annual increase of 7.72% (AAPC, 7.72%, 95% CI, 2.19%–16.47%). The age‐adjusted suicide rate among Whites did not change (2.0 deaths per 100,000 to 2.0 deaths per 100,000), with an average annual increase of 0.79% (AAPC, 0.79%, 95% CI, −1.67%–3.49%). The age‐adjusted suicide rate among other races increased by 181.8% (1.1 deaths per 100,000 to 3.1 deaths per 100,000), with an average annual increase of 7.59% (AAPC, 7.59%, 95% CI, −0.83%–21.47%) (Table 2).

The trends in Blacks consisted of 1 segment; a significant APC of 7.72% (95% CI, 2.19%–16.47%) in the first segment (2012–2022). The trends in Whites consisted of 2 segments; a significant APC of 6.6% (95% CI, 2.66%–20.76%) in the first segment (2012–2017), and a significant APC of −4.74% (95% CI, −15.42% to −0.10%) in the second segment (2017–2022). The trends in other races consisted of 1 segment; a significant APC of 7.59% (95% CI, −0.83%–21.47%) in the first segment (2012–2022). (Table 2).

### 3.5 Suicide by age groups

From 2012 to 2022, the age‐adjusted mortality rate among the 5–14‐year age group increased by 320% (0.5 deaths per 100,000 to 2.1 deaths per 100,000), with an average annual increase of 5.47% (AAPC, 5.47%, 95% CI, −0.23%–14.33%). The age‐adjusted mortality rate among the 15–24‐year age group increased by 14.9% (9.4 deaths per 100,000 to 10.8 deaths per 100,000), with an average annual increase of 2.52% (AAPC, 2.52%, 95% CI, −0.10%–6.64%). The age‐adjusted mortality rate among the 25–34‐year age group increased by 35.2% (16.5 deaths per 100,000 to 22.3 deaths per 100,000), with an average annual increase of 5.09% (AAPC, 5.09%, 95% CI, 2.40%–8.65%). The age‐adjusted mortality rate among the 35–44‐year age group declined by 20.1% (22.4 deaths per 100,000 to 17.9 deaths per 100,000), with an average annual decline of 0.05% (AAPC, −0.05%, 95% CI, −3.57%–3.98%). The age‐adjusted mortality rate among the 45–54‐year age group declined by 16.1% (22.4 deaths per 100,000 to 18.8 deaths per 100,000), with an average annual decline of 1.03% (AAPC, −1.03%, 95% CI, −4.81%–2.67%). The age‐adjusted mortality rate among the 55–64‐year age group decreased by 5.73% (15.7 deaths per 100,000 to 14.8 deaths per 100,000), with an average annual decline of 1.72% (AAPC, −1.72%, 95% CI, −7.09%–3.38%). The age‐adjusted mortality rate among the 65–74‐year age group increased by 39.7% (11.6 deaths per 100,000 to 16.2 deaths per 100,000), with an average annual increase of 3.13% (AAPC, 3.13%, 95% CI, −2.33%–9.88%). The age‐adjusted mortality rate among the 75–84‐year age group declined by 9.68% (21.7 deaths per 100,000 to 19.6 deaths per 100,000), with an average annual increase of 2.74% (AAPC, 2.74%, 95% CI, −1.02%–7.36%). The age‐adjusted mortality rate among 85 years and older age group increased by 40.8% (23.3 deaths per 100,000 to 32.8 deaths per 100,000), with an average annual increase of 2.16% (AAPC, 2.16%, 95% CI, −7.41%–12.65%) (Table 2).

The trends in the 5–14‐year age group consisted of 1 segment, a significant APC of 5.47% (95% CI, −0.23%–14.33%) in the segment (2012–2022). The trends in the 15–24‐year age group consisted of 1 segment, a significant APC of 2.52% (95% CI, −0.10%–6.64%) in the  segment (2012–2022). The trends in the 25–34‐year age group consisted of 1 segment, a significant APC of 5.09% (95% CI, 2.40%–8.65%) in the segment (2012–2022). The trends in the 35–44‐year age group consisted of 1 segment, a significant APC of −0.05% (95% CI, −3.57%–3.98%) in the segment (2012–2022). The trends in the 45–54‐year age group consisted of 1 segment, a significant APC of −1.03% (95% CI, −4.81%–2.67%) in the segment (2012–2022). The trends in the 55–64‐year age group consisted of 1 segment, a significant APC of −1.72% (95% CI, −7.09%–3.38%) in the segment (2012–2022). The trends in the 65–74‐year age group consisted of 1 segment, a significant APC of 3.13% (95% CI, −2.33%–9.88%) in the segment (2012–2022). The trends in the 75–84‐year age group consisted of 1 segment, a significant APC of 2.74% (95% CI,‐1.02%–7.36%) in the segment (2012–2022). The trends in the 85‐year age group and older group consisted of 1 segment, a significant APC of 2.16% (95% CI, −7.41%–12.65%) in the segment (2012–2022) (Table 2).

## 4 DISCUSSION

Among pediatric and adolescent patients aged 15–24 years, age‐adjusted suicide rates increased by 14.9% between 2012 and 2022. Suicide rates were highest in males, and Whites. Pediatric and adolescent patients aged 15–24 years had the fifth highest suicide rate among all age groups. These findings are identical to national statistics regarding suicide.^8^ Males tend to have a higher rate of suicide due to having more access to firearms, which are the most common means of suicide.^9^ However, the direction of the trends for age‐adjusted suicide rates differ between Mississippi and the national average. In Mississippi, there are upward trends for females, Blacks, and other races. There have been other studies with similar findings. Several studies have shown increasing rates of suicide in Black youth.^10^, ^11^ Other studies have also shown that other racial minorities, such as Asian or Pacific Islanders, and American Indian or Alaska Natives have shown increasing rates of suicide compared to Whites.^12^, ^13^ There have also been similar findings for studies evaluating suicide and gender. A study by the American Foundation for Suicide Prevention revealed that females attempted suicide 1.86 times more than males.^14^ Other studies have also found increasing rates of suicide in females.^15^, ^16^ Although males had an upward trend from 2012 to 2015, they had a downward trend from 2015 to 2022. Whites also experienced similar trends. Although whites had an upward trend from 2012 to 2017, they had a downward trend from 2017 to 2022. These findings highlight a need for awareness, education, and increasing availability of mental health services among young Mississippians to eliminate the risk of suicide. Medical and public health practitioners have attributed various risk factors to suicide, including mental illness/substance use, intrapersonal, interpersonal, and community factors, social media use and cyberbullying, and access to firearms.^9^ In Mississippi, the high rates of suicide among pediatric and adolescent patients aged 15–24 years tends to be associated with similar risk factors. However, one of the major risk factors for youth in Mississippi is access to firearms. About 72% of the reported suicide cases in youth are caused by firearms in Mississippi.^4^ The increased risk of suicide among youth, especially among Blacks, females, and other minority racial groups, that are associated with various risk factors are due to poor coping strategies in youth. Youth have not developed the ability to cope positively with stressful situations, which leads to the use of negative coping strategies that can cause harm, such as drug use and suicide.

As previously discussed, there was a recent decrease in age‐adjusted suicide rates among Whites and males aged 15–24 years in Mississippi. These findings could be attributed to local and national programs, and trainings that target and bring awareness to suicide in Whites and males. These campaigns have been launched both nationally and locally to target more at‐risk groups. However, there needs to be a shift in focus as rates and trends in suicide rise in racial minorities and females to prevent future racial and gender disparities in suicide mortality.

Based on the findings, Mississippi needs more initiatives aimed toward equitable preventing suicide among youth representing all genders and racial groups and the implementation of gun control policies. By creating equitable prevention initiatives and implementing gun control, Mississippi could tremendously benefit and improve health outcomes within the state. It would also allow Mississippi to possibly reduce suicide trends among the youth.

This study has some limitations. First, only reported cases of suicide were included in the study, which may have left out youth with unreported suicide deaths. Second, based on the nature of the study, there is a low capacity to estimate associations. Third, there is a limitation for the study's timeframe due to the unavailability of data after 2022, which is why the study focused primarily on findings between 2012 and 2022. Fourth, the dataset does not allow the analysis of different ages for males, females, and different races. Finally, the analysis was only stratified by age, race, and sex due to the lack of data on other groups, such as LGBTQIA2S+ within the statewide database.

The major strength of this study was its' use of surveillance data from Vital Statistics within the Mississippi State Department of Health. The study is also sound because it focuses on analyzing trends and changes over periods of time. The reports and data from the MSTAHRS database are reliable and consistent with findings in the United States.^17^ The results are also generalizable to Mississippi and other states within the southern region.

## 5 CONCLUSION

From 2012 to 2022, there is an upward trend of suicide in Mississippi for females, Blacks, and other races. Whites and males have significantly higher suicide rates than any other race, and gender. Youth aged 15–24 years also have the fifth highest rate of suicide among all age groups. Suicide is also the third leading cause of death in pediatric and adolescent patients aged 15–24 years. The access to firearms is a major factor in suicide‐related deaths among youth in Mississippi. Due to the significant impact of suicide, it is important that Mississippi initiates more programs that will promote equitable prevention measures that educate communities and bring awareness to suicide in the state to ensure that all Mississippians, especially at‐risk groups are able to improve their mental health outcomes and reduce the risk of suicide.^18^, ^19^, ^20^, ^21^, ^22^, ^23^, ^24^ The implementation of gun control policies would also make a significant impact in reducing the risk of suicide in the state.^25^, ^26^, ^27^ Through the implementation of gun control policies, Mississippi could reduce access to firearms that contribute to more than half of all suicides in youth. Therefore, the impact of suicide, the need for the implementation of gun control policies, and the need for equitable prevention efforts within the state elicit a need for a strong and inclusive public health response and further statistical analyses.

## AUTHOR CONTRIBUTION

The authors confirm contribution to the paper as follows: study conception and design: **E.J.**; data collection: **E.J.**; analysis interpretation of results: **E.J.**; draft manuscript preparation: **E.J.** The author reviewed the results and approved the final version of the manuscript.

## CONFLICT OF INTEREST STATEMENT

The author declares that there is no conflicts of interest.

## ETHICS STATEMENT

Ethical approval for this study was obtained from the Mississippi State Department of Health, Vital Records, and Public Health Statistics Committee (April 25, 2024).

## INSTITUTIONAL REVIEW Board STATEMENT

Not applicable.

## INFORMED CONSENT STATEMENT

Not applicable.

## Data Availability

The data that support the findings of this study are available in Mississippi Statistically Automated Health Resource System at https://mstahrs.msdh.ms.gov/forms/morttable.html. These data were derived from the following resources available in the public domain: Mississippi Statistically Automated Health Resource System, https://mstahrs.msdh.ms.gov/forms/morttable.html.
